# Epigenetic silencing of *PDLIM4* promotes colorectal cancer by impairing BTF3 autophagic degradation and activating PI3K/Akt signalling

**DOI:** 10.1002/ctm2.70882

**Published:** 2026-10-30

**Authors:** Jiaxing Wang, Chuan‐Guo Guo, Lingyu Qi, Songyuan Hou, Boshen Lin, Tong Ye, Ye Zong, Shutian Zhang, Nan Zhang, Peng Li, Si Liu

**Affiliations:** ^1^ Department of Gastroenterology, Beijing Friendship Hospital, Capital Medical University; State Key Laboratory of Digestive Health; National Clinical Research Center for Digestive Disease Beijing Key Laboratory of Early Gastrointestinal Cancer Medicine and Medical Devices Beijing China

**Keywords:** BTF3, colorectal cancer, DNA methylation, PDLIM4, PI3K/Akt pathway

## Abstract

**Background:**

Epigenetic silencing of tumour suppressor genes is a pivotal early event in colorectal cancer (CRC) pathogenesis. PDZ and LIM Domain 4 (PDLIM4) is a member of the PDZ‐LIM family with context‐dependent roles in tumour progression across different malignancies. However, its specific role and epigenetic regulation in colorectal cancer (CRC) remain elusive.

**Methods:**

We integrated multi‐omics datasets and clinical cohorts to evaluate PDLIM4 expression and promoter methylation. Stool DNA was analyzed to assess the feasibility of non‐invasive detection of PDLIM4 promoter methylation. PDLIM4 knockout and overexpression cell lines were constructed to perform functional assays including proliferation, apoptosis, and stemness. In vivo experiments were validated utilizing xenograft mice or an azoxymethane (AOM)/ dextran sulphate sodium salt (DSS) CRC mouse model with intestinal epithelial cell‐specific overexpression of PDLIM4. Mechanistic investigations were conducted using coimmunoprecipitation coupled with mass spectrometry, RNA sequencing, ChIP‐qPCR and pharmacological rescue experiments.

**Results:**

We identified a progressive loss of PDLIM4 protein along the normal‐adenoma‐carcinoma sequence, driven by DNMT3A‐mediated promoter hypermethylation, and lower PDLIM4 expression was associated with adverse patient prognosis. PDLIM4 methylation was also significantly increased in stool DNA from CRC patients compared with healthy controls. Mechanistically, genetic ablation of PDLIM4 significantly blunted the therapeutic efficacy of the DNA demethylating agent 5‐Azacytidine against CRC. Functionally, PDLIM4 restoration suppressed CRC cell proliferation and stemness while enhancing apoptosis in vitro, and reduced tumour growth in both AOM/DSS and xenograft models in vivo. At the molecular level, PDLIM4 interacted with BTF3 and promoted its autophagic‐lysosomal degradation, thereby limiting PI3K/Akt signalling, with NTRK2 identified as a potential transcriptional target linking BTF3 to PI3K/Akt activation.

**Conclusion:**

DNMT3A‐mediated silencing of PDLIM4 is an early epigenetic event that facilitates CRC progression. PDLIM4 suppresses tumour growth by promoting BTF3 degradation, thereby restricting PI3K/Akt signalling. Thus, PDLIM4 methylation may have potential as a biomarker for early CRC detection.

**Key points:**

- DNMT3A‐mediated PDLIM4 promoter hypermethylation is an early event in colorectal tumorigenesis.
- Restoring PDLIM4 suppresses CRC proliferation and stemness while promoting apoptosis in vitro and in vivo.
- PDLIM4 promotes the autophagic‐lysosomal degradation of BTF3 and restrains PI3K/Akt signalling.
- Stool DNA analysis supports the potential of PDLIM4 methylation as a non‐invasive CRC biomarker

## Graphical abstract

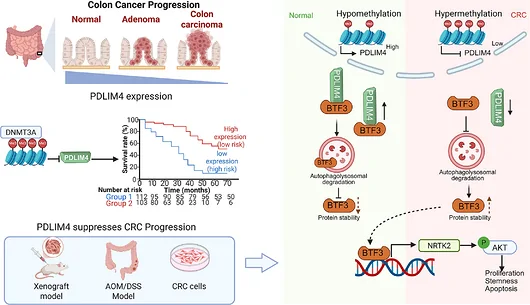

## 1 BACKGROUND

Colorectal cancer (CRC) remains a major cause of cancer mortality worldwide.^1^ Colorectal adenoma represents the primary precancerous lesion for CRC, and the ‘adenoma‐carcinoma’ sequence constitutes its most significant developmental pathway, accounting for approximately 60–70% of cases.^2^ The stepwise transition from normal epithelium to adenoma and ultimately to invasive carcinoma provides a critical window for early detection and intervention. However, the exact molecular events driving this early malignant transformation remain incompletely understood.

Epigenetics, particularly DNA promoter hypermethylation, is universally driving the transcriptional silencing of critical tumour suppressor genes (TSGs) during colorectal tumorigenesis. Nowadays, DNA methylation biomarkers have emerged as a non‐invasive, effective approach in detecting early‐stage malignancies.^3^ Circulating SEPT9 and faecal NDRG4/BMP3 methylation panels are already approved by the FDA and actively utilized for early disease detection.^4^, ^5^ While these markers are highly sensitive for cancer, it shows limited sensitivity in detecting adenoma. The specific hypermethylated TSG that actively drives the adenoma‐to‐carcinoma transition remains poorly defined. Therefore, identifying key methylated events that contribute to adenoma and CRC offers a potential epigenetic therapy target, with the goal of restoring tumour suppressor gene function and bettering patient health.

PDZ‐LIM domain family comprises 10 proteins (PDLIMs), which function as scaffold proteins that coordinate protein‐protein interactions, cytoskeletal anchoring, and signal transduction.^6^ Members of this family have been implicated in various cancer‐related processes, while displaying striking heterogeneity across different cancer types.^7^, ^8^, ^9^, ^10^ For example, PDLIM1 has been shown to suppress hepatocellular carcinoma metastasis by activating Hippo signalling.^11^ PDLIM4 has also been implicated in tumour progression, with context‐dependent roles reported across different malignancies. Extensive epigenetic profiling has further identified PDLIM4 as a frequent target of promoter hypermethylation across various malignancies.^12^, ^13^, ^14^, ^15^ However, the functional consequences of its epigenetic silencing in colorectal cancer (CRC) remain strikingly unexplored. Although initial evidence of PDLIM4 hypermethylation was observed in colon‐derived systems, its specific functional role in colorectal cancer remains poorly understood. Therefore, defining the specific biological functions and downstream molecular networks of PDLIM4 is critical to clarify its role in early colorectal tumorigenesis.

In the present study, we identify *PDLIM4* as a tumour suppressor gene whose epigenetic silencing via DNA hypermethylation constitutes an early event in adenoma‐to‐CRC development. Clinically, the downregulation of PDLIM4 is associated with poor patient prognosis. Using an AOM/DSS‐induced CRC model, we demonstrate that PDLIM4 restoration significantly suppresses tumour progression and promotes cancer cell apoptosis. Mechanistically, PDLIM4 interacts with BTF3 and promotes its autophagic‐lysosomal degradation, thereby attenuating oncogenic PI3K/Akt signalling. Together, these findings identify PDLIM4 as an epigenetically regulated tumour suppressor in CRC and highlight its potential biological and translational relevance.

## 2 METHODS

### 2.1 Cell culture

The human CRC cell lines (HCT15, SW480, HT‐29, HCT116, Caco‐2 and RKO) and normal colorectal epithelial cells (NCM460) were utilized in this study. SW480 and HCT15 cells were grown in RPMI 1640 (#03.4007C, Eallbio) medium supplemented with 10% fetal bovine serum (FBS, #03.C16001DC, Eallbio), while HT‐29 and HCT116 were maintained in McCoy's 5A medium with 10% FBS. NCM460 and RKO cell lines were cultured using Dulbecco's modified Eagle's medium (DMEM, #03.1002C, Eallbio) with 10% FBS, whereas the Caco‐2 line was kept in minimum essential medium (MEM, #03.10002C, Eallbio) with 20% FBS. Cells were cultured at 37°C with 5% CO_2_ in the appropriate medium containing 1% penicillin‐streptomycin.

### 2.2 Human tumour samples

Immunohistochemical (IHC) analysis of PDLIM4 expression was performed using formalin‐fixed, paraffin‐embedded specimens from 15 colorectal carcinomas, 29 adenomas, and 15 normal colonic tissues. Furthermore, eight pairs of human colorectal cancer tissues were obtained from patients at the Beijing Friendship Hospital's Endoscopy Center, while 20 stool samples were gathered from individuals either undergoing check‐ups or diagnosed with colorectal cancer at the same facility. This research received approval from the Beijing Friendship Hospital Institutional Ethics Committee (approval number 2018‐P2‐058‐05).

### 2.3 Animal studies

Specific‐pathogen‐free housing at the Capital Medical University animal facility was used for C57BL/6J‐WT, C57BL/6J‐villin cre (both 5–8 weeks old), and BALB/c nude mice (4–5 weeks old) acquired from Beijing Vital River Laboratory Animal Technology Co., Ltd. (1) AOM/DSS‐induced colorectal adenoma and CRC model. Mice were identified by toe clipping and randomly assigned to experimental groups by an independent investigator. Colorectal tumorigenesis was induced by intraperitoneal administration of AOM (10 mg/kg; A5486, Sigma‐Aldrich), followed by 1% DSS (#0216011080, MP Biomedicals) in the drinking water for 7 days per cycle, for either one or three cycles. Mice were sacrificed at week 15. For intestinal PDLIM4 overexpression, the AAV‐DIO‐PDLIM4 vector was purchased from OBiO Technology and administered to *Villin‐Cre* mice as described above. (2) The subcutaneous xenograft model. Human CRC cell line HCT15 (5 × 10^6^ cells in 100 µL PBS) or SW480 (8 × 10^6^ cells in 100 µL PBS) were subcutaneously injected into the left axilla (5 mice per group). Tumour dimensions were recorded at 2‐day intervals, and tumour volume was estimated according to the formula: length × width^2^/2, as previously reported.^16^ For the in vivo experiments, tumour volume measurements and IHC quantification were performed by investigators blinded to group allocation. Coded cage labels were used during tumour measurements, and tissue sections were coded before IHC quantification. At the experimental endpoint, tumours were excised, photographed, and weighed. All animal procedures were reviewed and approved by the Ethics Committee of Capital Medical University (approval number AEEI‐2025‐1130).

### 2.4 Methylation‐specific PCR (MSP) and real‐time quantitative MSP (Q‐MSP)

MSP and Q‐MSP were used to assess PDLIM4 promoter methylation.^17^ Following the protocols provided by the manufacturers, genomic DNA was isolated from tissue and cell cultures with the QIAamp DNA Mini Kit (#51304, Qiagen) and from faecal matter using a specific stool DNA extraction kit (#116547050, MP Biomedicals). Afterwards, bisulphite treatment was performed on the DNA using the EpiArt DNA Methylation Bisulfite Kit V2 (EM102, Vazyme) according to the provided instructions, carefully. For Q‐MSP, we used the methylated primer and input control (ACTB gene) for PCR. The percentage of methylated reference (PMR) value of PDLIM4 was used to represent gene methylation level.^18^ The PMR was calculated using the 100 × 2^−ΔΔCt^ quantification approach, where ΔΔCt = [(Ct_PDLIM4 − Ct_ACTB)_sample − (Ct_PDLIM4 − Ct_ACTB)_fully methylated control].^19^ For conventional MSP, PCR products amplified with methylated (M) and unmethylated (U) primers were separated by agarose gel electrophoresis. Band intensities were quantified using ImageJ, and the relative methylation ratio was calculated as M/(M + U). Primers are shown in Table S1.

### 2.5 Immunohistochemistry (IHC) staining assays

The tissue samples were fixed for 24 h in 4% paraformaldehyde, followed by dehydration in ethanol and paraffin embedding. Endogenous peroxidase was neutralized with 3% H_2_O_2_ for 10 min after high‐pressure antigen retrieval (PV‐6000, ZSGB‐BIO). The sections were blocked with goat serum (ZLI‐9022, ZSGB‐BIO) for 1 h, then incubated with primary antibodies overnight at 4°C. After a 1.5 h incubation with secondary antibodies at room temperature, the signal was developed using a diaminobenzidine (DAB) kit (ZLI‐9019, ZSGB‐BIO) and counterstained with haematoxylin. This procedure followed established methods,^20^, ^21^, ^22^ and the primary antibody details were available in Table S2.

High‐resolution images were acquired using an inverted microscope (DMiL, Leica) under consistent imaging settings. IHC staining was quantified using ImageJ software. For Ki‐67 staining, the relevant image channel was extracted using the Split Channels function, and the same threshold was applied to all images within the same experiment. Ki‐67‐positive nuclei and total nuclei were counted, and the percentage of Ki‐67‐positive nuclei was calculated. For cytoplasmic proteins, colour deconvolution was performed to separate the haematoxylin and DAB signals, and the DAB‐positive area fraction within the defined region of interest was measured. All images were acquired and analyzed using identical parameters across groups to ensure comparability.

### 2.6 Lentivirus and transfection

To create PDLIM4‐deficient SW480 cells, the researchers employed the lentiCRISPR v2 system. A pair of complementary oligonucleotides targeting PDLIM4 was synthesized, annealed, and cloned into the lentiCRISPR v2 vector (forward: 5′‐CACCGTGCTGGCAGCAAGGCTGCAT‐3′; reverse: 5′‐AAACATGCAGCCTTGCTGCCAGCAC‐3′). Simultaneously, a PCDH‐PDLIM4‐3×FLAG construct was developed to facilitate stable protein overexpression. These vectors were then co‐transfected with essential packaging plasmids into 293T cells to generate lentiviruses, which were subsequently used to infect colorectal cancer cells. Subsequent to puromycin (S7417, Selleck) selection, Western blotting was performed to validate the resulting PDLIM4 knockout and overexpression levels.

For transient overexpression of BTF3, a plasmid encoding HA‐tagged BTF3 (pcDNA3.1‐BTF3‐HA‐C) was generated. The mCherry‐EGFP‐LC3 dual‐fluorescence reporter plasmid was purchased from Youbio and used to assess autophagic flux. The sequences of the small interfering RNAs (siRNAs) used in this study are listed in Table S3. Transient transfection was performed using MegaTran 2.0 (TT210003, OriGene) for plasmids or Lipofectamine 2000 (11668019, Thermo Fisher Scientific) for siRNAs, according to the manufacturers’ instructions. Cells were treated 48 h after transfection and subsequently collected for further analysis.

### 2.7 Immunofluorescence (IF) microscopy

Following two washes with 1× PBS, cells were fixed for 30 min. The samples were then permeabilized with 0.3% Triton X‐100 (#9036‐19‐5, Sigma) in PBS for 20 min and blocked with 5% BSA (WB6504, NCM Biotech) in PBS for 1 h. After primary antibodies were incubated at 4°C overnight, Alexa Fluor 488 (A32723, Invitrogen)‐ or Alexa Fluor 594 (A11012, Invitrogen)‐conjugated secondary antibodies were used to stain the cells for 1 h at room temperature in dark conditions. For PDLIM4–BTF3 co‐localization analysis, cells were co‐transfected with PDLIM4‐Flag and BTF3‐HA plasmids before immunofluorescence staining. For assessment of autophagic flux, cells were transfected with the mCherry‐EGFP‐LC3 dual‐fluorescence reporter and treated as indicated. DAPI (SC‐24941, Santa Cruz) or Hoechst 33342 (C0031, SolarBio) was used to counterstain nuclei for 10 min. High‐resolution images were captured using a confocal microscope (FV1200, Olympus) with consistent imaging parameters, including laser intensity and exposure time, across all groups.

### 2.8 TUNEL staining

The presence of apoptotic cells in tissue sections was determined using a commercial assay kit (E‐CK‐A322, Elabscience) in accordance with the standard protocol. After the sections were deparaffinized and rehydrated, they were treated with diluted proteinase K for 30 min at 37°C and then incubated with the TdT enzyme reaction mixture for 2 h at 37°C. The slides were eventually stained with DAPI for 10 min at room temperature, and fluorescence images were analyzed using ImageJ. The percentage of apoptotic cells was computed by dividing the number of TUNEL‐positive cells by the total count of DAPI‐positive nuclei in the same field of view.

### 2.9 Cell counting kit‐8 (CCK‐8) assay

Roughly 1000 cells were placed into each well of a 96‐well plate, and at different intervals after seeding, the wells received fresh medium with 10% CCK8 substrate (#03.17002DC, Eallbio). The cells were maintained at 37°C for 2 h. Cell proliferation was then quantified based on the absorbance values (450 nm).

### 2.10 5‐Ethynyl‐2’‐deoxyuridine (EdU) staining

Following overnight culture of 1 × 10^5^ cells per well in 12‐well plates, an EdU‐594 kit (C0078, Beyotime) was used for staining. This process included a 2 h incubation in 20 µM EdU at 37°C, PBS elution, 4% PFA fixation, and 0.3% Triton X‐100 permeabilization. Cells then underwent a 0.5 h click reaction in the dark. For nuclear visualization, cells were incubated with 1× Hoechst 33342 for 10 min in the dark and rinsed with PBS. Fluorescence images were subsequently acquired using a fluorescence microscope.

### 2.11 Colony formation assay

In six‐well plates, transfected or drug‐treated cells were distributed at 300–500 cells per well and grown for 2 weeks at 37°C. Once fixed and stained for 30 min with 0.3% crystal violet, the resulting colonies were captured using a colony counter and analyzed with ImageJ software for quantification.

### 2.12 Flow cytometry

Apoptosis was assessed by flow cytometry using the PE Annexin V apoptosis detection kit with 7‐AAD (#640934, BioLegend). Treated cells were resuspended in 100 µL Binding Buffer. PE Annexin V and 7‐AAD viability staining solution were used to distinguish viable, early apoptotic, and late apoptotic/dead cells. Analyses were performed using a high‐efficiency BD FACS Celesta cell analyzer (BD Biosciences).

### 2.13 Tumoursphere‐formation assay

The steps of the tumoursphere‐formation experiment were performed as in previous studies.^23^, ^24^ CRC cells were plated at an ideal density into ultra‐low attachment plates (#3471, Corning). Seven days afterwards, tumourspheres measuring 50 µm or larger were quantified using ImageJ software.

### 2.14 Co‐immunoprecipitation (CO‐IP)

RKO cells were separately transfected with PDLIM4‐Flag or BTF3‐HA plasmids. Cells were collected 48 h post‐transfection and subsequently lysed in the appropriate lysis buffer (P0013, Beyotime Biotechnology) supplemented with protease inhibitors (#4693116001, Roche). Samples were centrifuged at 12 000 rpm for 15 min at 4°C, after which the supernatants were collected. For reciprocal Co‐IP, lysates from PDLIM4‐Flag‐transfected cells were incubated with 25 µL anti‐Flag Nanobody Magarose Beads (KTSM1338, AlpaLifeBio) to immunoprecipitate PDLIM4, followed by Western blot detection of BTF3. Conversely, lysates from BTF3‐HA‐transfected cells were incubated with 25 µL anti‐HA Nanobody Magarose Beads (KTSM1335, AlpaLifeBio) to immunoprecipitate BTF3, followed by Western blot detection of PDLIM4. The beads were incubated overnight at 4°C and washed three times with PBST (PBS supplemented with 0.05% Tween‐20). Bound proteins were then eluted in loading buffer and analyzed by Western blotting. Appropriate negative controls were included in parallel.

### 2.15 RNA isolation and quantitative real‐time polymerase chain reaction (qRT‐PCR)

TRIzol reagent (#15596018, Thermo Fisher) was used for total RNA isolation, followed by reverse transcription with PrimeScript RT Master Mix (RR036A, TaKaRa). For qRT‐PCR, a Q7 real‐time thermal cycler was employed with the specific primers provided in Table S4. Relative expression values were derived using the 2^−ΔΔC^ *^t^* technique after normalizing mRNA levels to the GAPDH control.

### 2.16 Protein isolation and Western blot (WB)

To assess protein expression, Western blotting was performed. Cells were lysed using RIPA buffer (P0013B, Beyotime Biotechnology). Following SDS‐PAGE separation, proteins were transferred onto PVDF membranes (ISEQ00010, Merck Millipore). Blocked membranes were exposed to the appropriate primary antibodies and subsequently to HRP‐conjugated secondary antibodies. The resulting bands were detected with a Bio‐Rad imaging system, and ImageJ software was used for quantification. Primary antibodies used for WB were shown in Table S2.

### 2.17 Chromatin immunoprecipitation (CHIP)

ChIP was performed using an antibody against DNMT3A or BTF3, with non‐specific IgG serving as a negative control. ChIP experiments were performed with a ChIP Assay Kit (P2078, Beyotime Biotechnology) following the manufacturer's protocol. The precipitated DNA was subjected to qRT‐PCR and agarose gel electrophoresis. Three pairs of ChIP‐qPCR primers overlapping the promoter region of the human PDLIM4 or NTRK2 gene were designed, as detailed in Table S5.

### 2.18 Reagents

Cycloheximide (CHX, HY‐12320), chloroquine (CQ, HY‐17589A), ammonium chloride (NH_4_Cl, HY‐Y1269), Z‐VAD‐FMK (HY‐16658B) and LY294002 (HY‐10108) were obtained from MedChemExpress (MCE). 3‐Methyladenine (3‐MA, S2767), 5‐azacytidine (5‐AZA, S1782) and MG132 (S2619) were obtained from Selleck.

### 2.19 Statistical analysis

For omics analyses, including RNA‐seq and DNA methylation array data, adjustment for multiple comparisons was performed to control the false discovery rate (FDR). For RNA‐seq data, *p*‐values were adjusted for multiple comparisons using the Benjamini–Hochberg method, with |log_2_FC| ≥ 1.0 and FDR < .05 used as the thresholds for differential expression. For DNA methylation array data, differential methylation analysis was performed using the limma package with empirical Bayes moderation, followed by Benjamini–Hochberg correction. CpG sites with Δβ ≥ .2 and FDR < .05 were considered differentially methylated.

All remaining statistical analyses were conducted with GraphPad Prism 9 (GraphPad Software, La Jolla, CA, USA). Data are presented as mean ± SEM unless otherwise specified. Sample sizes are indicated in the corresponding figure legends. Normality and homogeneity of variance were assessed before parametric analyses. For two independent groups, normally distributed data meeting parametric assumptions were compared with a two‐sided unpaired Student's *t*‐test; otherwise, a Mann–Whitney *U* test was performed. For paired samples, a paired two‐tailed Student's *t*‐test or Wilcoxon matched‐pairs signed‐rank test was used as appropriate. For analyses involving three or more groups, one‐way ANOVA followed by Dunnett's test was used when each group was compared with the same control. For analyses with two independent factors, two‐way ANOVA followed by Tukey's post hoc test was applied, and interaction effects were evaluated where appropriate. Longitudinal tumour growth was analyzed using two‐way repeated‐measures ANOVA. Spearman's rank correlation was applied to assess non‐parametric associations. A two‐sided *p*‐value < .05 was considered statistically significant.

## 3 RESULTS

### 3.1 Epigenetic silencing of *PDLIM4* is associated with colorectal cancer progression and adverse prognosis

To identify critical epigenetically driven TSGs during CRC development, we performed an integrated analysis of public databases. Firstly, the hypermethylated genes were integrated with downregulated genes by analyzing the TCGA database (Figure 1A). Next, the above intersection was crossed with hypermethylated genes (GSE36380) and downregulated genes in colorectal adenomas (GSE89076) (Figure 1B). The results showed that seven genes were consistently downregulated in both colorectal adenomas and carcinomas due to promoter hypermethylation (Figure S1A). By evaluating their correlation with DNA methylation status and their prognostic significance via survival analysis, we ultimately pinpointed *PDLIM4* as the most promising candidate (Figure S1A–C). Subsequent validation using independent GEO datasets confirmed that PDLIM4 expression was significantly downregulated in adenomas, accompanied by an elevation in its promoter methylation levels (Figure 1C,D). This trend persisted in CRC tissues, where PDLIM4 expression remained markedly decreased and exhibited a robust negative correlation with its promoter methylation degree (Figure 1E,F).

**FIGURE 1:**
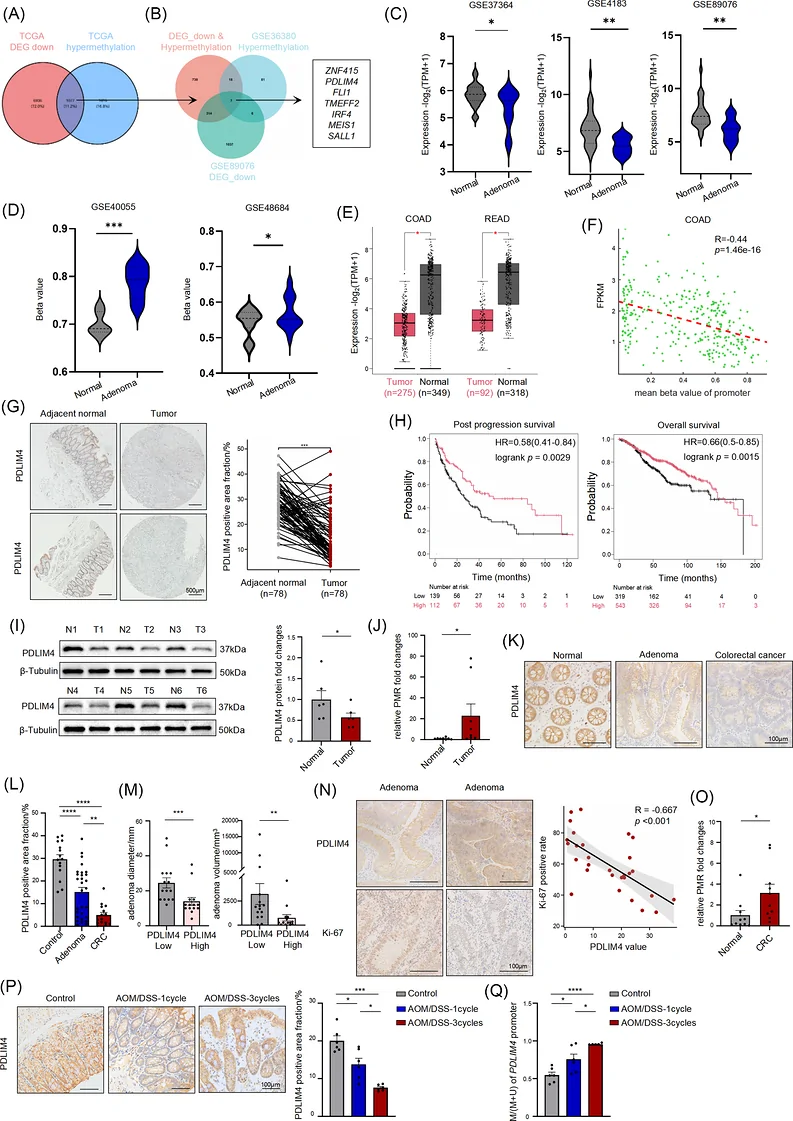
PDLIM4 is progressively silenced by promoter hypermethylation during colorectal tumorigenesis and is associated with adverse clinical outcomes. (A) Venn diagram illustrating the intersection of downregulated and hypermethylated genes associated with CRC from the TCGA database. (B) Identification of key candidate genes via the intersection of hypermethylated genes (GSE36380), downregulated genes in colorectal adenomas (GSE89076), and the overlapping genes identified in (A). (C and D) Violin plots depicting *PDLIM4* expression levels (C) and promoter methylation levels (D) across normal mucosa and adenoma from representative GEO datasets. (E) Comparison of *PDLIM4* expression levels between matched tumour and normal tissues using the TCGA database. (F) Spearman correlation between *PDLIM4* promoter methylation and FPKM of *PDLIM4* in colon adenocarcinoma (COAD) from the DNA Methylation Interactive Visualization Database (DNMIVD). (G) Immunohistochemistry (IHC) evaluation of PDLIM4 expression in a human CRC tissue microarray (TMA) containing paired CRC and adjacent normal colon tissues from 78 patients. Scale bars = 500 µm. (H) Kaplan–Meier analysis of post‐progression survival (left) and overall survival (right) based on *PDLIM4* expression in CRC patients from the Kaplan–Meier Plotter database. (I, J) Western blot (WB) (I) and quantitative methylation‐specific PCR (Q‐MSP) (J) analysis of PDLIM4 expression and methylation status, respectively, in fresh CRC tissues (T) and paired normal tissues (N). (K and L) IHC assessment of PDLIM4 expression across normal mucosa (*n* = 14), adenoma (*n* = 29), and CRC (*n* = 15) tissues. Scale bars = 100 µm. (M) Quantitative analysis of adenoma size and volume stratified by PDLIM4 expression levels. (N) Representative IHC staining for PDLIM4 and Ki‐67 in human colorectal adenoma tissues. Scale bars = 100 µm; Scatter plots show the Spearman correlation between PDLIM4 expression and Ki‐67 proliferative index. (O) Q‐MSP analysis of PDLIM4 methylation status in stool from CRC patients (*n* = 10) and healthy controls (*n* = 10). (P) IHC analysis of PDLIM4 in AOM/DSS induced adenoma and CRC mouse models. Scale bars = 100 µm. (Q) Quantitative evaluation of *PDLIM4* promoter methylation levels [M/(M+U)] in murine intestinal tissues across distinct stages. Data are presented as mean ± SEM. **** *p *< .0001; *** *p *< .001; ** *p *< .01; * *p *< .05; n.s. *p *> .05.

To validate these findings in clinical cohorts, we assessed PDLIM4 protein levels using a human CRC tissue microarray (TMA). Immunohistochemistry (IHC) analysis confirmed that PDLIM4 expression was lower in tumour tissues than in paired adjacent normal mucosa (Figure 1G). Kaplan–Meier analysis showed that higher PDLIM4 expression was associated with improved overall survival, identifying PDLIM4 as a favourable prognostic indicator in CRC (Figure 1H). We next investigated the dynamic changes of PDLIM4 during the early stages of tumorigenesis. Western blotting of six paired clinical samples showed lower PDLIM4 protein levels in CRC tissues than in the corresponding normal tissues (Figure 1I). Consistently, PDLIM4 promoter methylation levels were significantly increased in CRC tissues (Figure 1J). Clinicopathological evaluation further delineated a progressive loss of PDLIM4 expression along the normal‐adenoma‐carcinoma sequence (Figure 1K,L). Notably, the loss of PDLIM4 in adenomas was strongly associated with increased tumour size (Figure 1M) and a higher degree of malignancy, as evidenced by enhanced Ki‐67 proliferative staining (Figure 1N). Importantly, PDLIM4 methylation levels were also significantly higher in stool DNA from CRC patients than in healthy controls (Figure 1O). Furthermore, a murine CRC model showed changes consistent with those observed in human samples (Figure S1D,E). Sequential histological and methylation analyses of mouse intestinal tissues confirmed a stepwise reduction in PDLIM4 expression, driven by progressive promoter hypermethylation during tumour advancement (Figure 1P,Q). Finally, we verified this epigenetic silencing in vitro; compared with normal intestinal epithelial cells (NCM460), PDLIM4 expression was profoundly decreased in a panel of CRC cell lines (Figure S1F,G), corresponding to an elevated promoter methylation status (Figure S1H).

### 3.2 PDLIM4 suppresses proliferation, apoptosis resistance, and stemness in colorectal cancer cells

To investigate the role of PDLIM4 in CRC progression in vitro, we generated PDLIM4‐knockout (PDLIM4‐KO) SW480 and HCT15 cells and established stable PDLIM4 overexpression in RKO and HCT15 cells, with successful genetic manipulation confirmed by Western blotting (Figure 2A; Figure S2A). CCK‐8, EdU incorporation, and colony formation assays collectively demonstrated that *PDLIM4* depletion markedly accelerated cell proliferation, whereas its ectopic overexpression exerted a profound inhibitory effect compared with their respective controls (Figure 2B–D; Figure S2B–D). Furthermore, flow cytometry analysis revealed that *PDLIM4* ablation significantly attenuated hydrogen peroxide‐induced apoptosis in SW480 cells. Conversely, *PDLIM4* overexpression sensitized RKO cells to apoptotic cell death (Figure 2E). These phenotypic observations were corroborated by Western blot analysis, which showed a decreased Bax/Bcl‐2 ratio and diminished cleaved caspase‐3 levels in *PDLIM4*‐KO SW480 cells, alongside an inverse molecular signature (a higher Bax/Bcl‐2 ratio together with increased cleaved caspase‐3 levels) in *PDLIM4*‐overexpressing RKO cells (Figure 2F). Beyond proliferation and survival, we further investigated the impact of PDLIM4 on CRC stemness. Tumoursphere formation assays revealed a striking increase in the number of spheres following *PDLIM4* knockout, which was accompanied by the upregulation of core stemness markers (CD133, OCT4, and Nanog) at both the mRNA and protein levels. Consistent with these findings, PDLIM4 restoration in RKO cells reduced sphere formation and lowered the expression of stemness‐associated markers (Figure 2G–I). Together, these in vitro findings establish PDLIM4 as a potent negative regulator of CRC malignancy.

**FIGURE 2:**
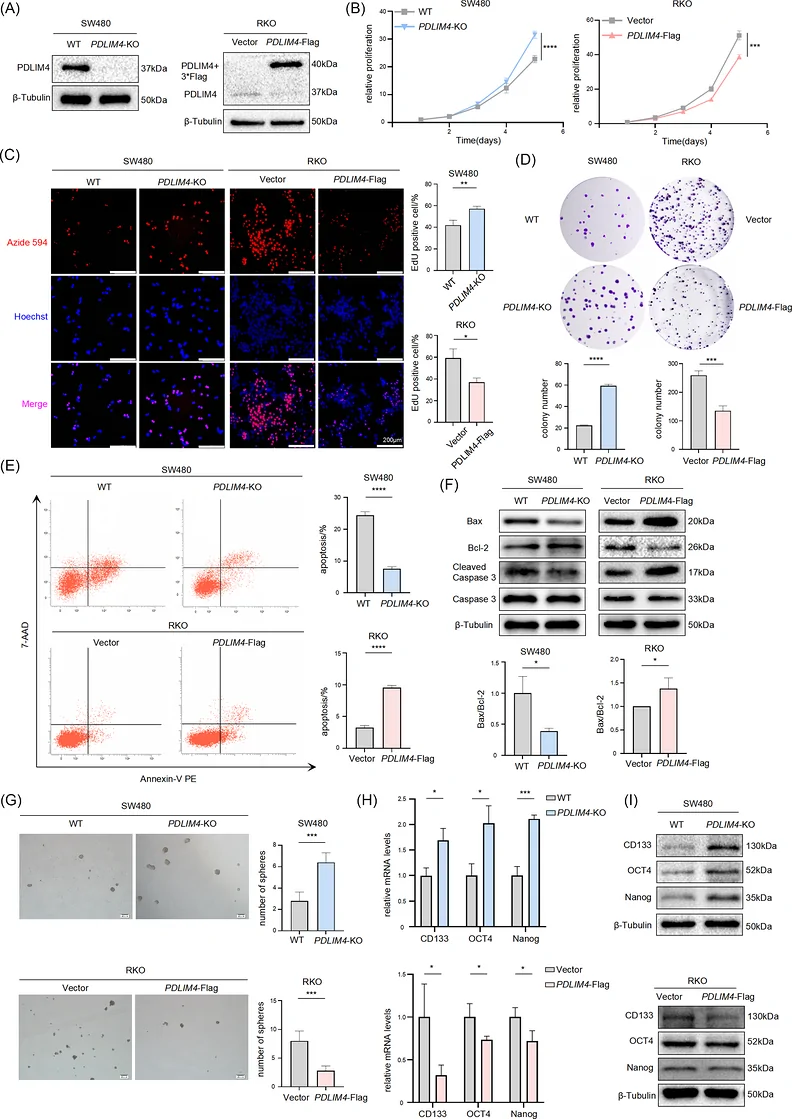
PDLIM4 suppresses the malignant phenotypes of CRC cells by restricting proliferation and stemness while sensitizing cells to apoptosis. (A) Western blot validation of CRISPR/Cas9‐mediated *PDLIM4* knockout (KO) in SW480 cells and stable *PDLIM4* overexpression (Flag‐tagged) in RKO cells. (B–D) Evaluation of cell proliferative capacity using CCK‐8 (B), EdU incorporation (C), and colony formation (D) assays in *PDLIM4*‐KO SW480 and *PDLIM4*‐overexpressing RKO cells, alongside their respective wild‐type controls. Scale bars = 200 µm. (E) Flow cytometric analysis of apoptosis induced by H_2_O_2_ treatment in the indicated CRC cell lines. The apoptosis rate was calculated based on the proportion of early apoptotic cells in the lower right quadrant (Annexin V^+^/7‐AAD^−^). (F) Western blot analysis of apoptosis‐associated proteins (Cleaved Caspase‐3, Bax, and Bcl‐2) following *PDLIM4* depletion or overexpression. (G) Representative images (left) and quantitative analysis (right) of tumoursphere formation assays assessing the stemness of *PDLIM4*‐KO SW480 and *PDLIM4*‐overexpressing RKO cells. Scale bars = 200 µm. (H, I) Quantitative RT‐PCR (H) and Western blot (I) analyses of stemness markers (CD133, OCT4, and Nanog) in the indicated CRC cell lines upon PDLIM4 modification. Data are presented as mean ± SEM. **** *p *< .0001; *** *p *< .001; ** *p *< .01; * *p *< .05; n.s. *p *> .05.

### 3.3 PDLIM4 restoration suppresses colorectal tumorigenesis in vivo

Having established PDLIM4 as a potent negative regulator of CRC malignancy in vitro, we next sought to determine its tumour‐suppressive efficacy in vivo. To achieve this, we generated a murine model with intestinal epithelial cell‐specific *PDLIM4* overexpression by administering AAV‐DIO‐PDLIM4 into *Villin‐Cre* mice (hereafter AAV‐*PDLIM4*) (Figure 3A). Western blot analysis confirmed significantly increased PDLIM4 protein expression in the colonic tissue of Cre^+^ AAV‐*PDLIM4* mice compared with AAV‐Ctrl mice, whereas no apparent increase was observed in the liver (Figure 3B). IHC analysis of three separate intestinal segments, including the distal, middle, and proximal colon, showed comparable PDLIM4 expression across these regions in AAV‐PDLIM4 mice, indicating broad intestinal restoration of PDLIM4 expression (Figure S3A). These mice were subsequently subjected to an AOM/DSS regimen to drive inflammation‐associated colorectal tumorigenesis. Strikingly, murine endoscopic evaluation revealed a lower tumour burden in AAV‐*PDLIM4* mice compared with control cohorts (Figure 3C). Upon harvest, macroscopic inspection confirmed that *PDLIM4*‐overexpressing mice developed significantly fewer and smaller intestinal tumours (Figure 3D). Furthermore, histological assessment via H&E staining demonstrated that PDLIM4 restoration markedly decreased the proportion of advanced carcinoma lesions (Figure 3E). Consistent with our in vitro findings, Ki‐67 and TUNEL staining of the murine tumour tissues showed that intestinal epithelial cell‐specific *PDLIM4* overexpression markedly suppressed tumour cell proliferation and promoted apoptosis, while correlation analyses further revealed a negative trend between PDLIM4 expression and Ki‐67 positivity and a positive trend between PDLIM4 expression and TUNEL positivity (Figure 3F,G; Figure S3B,C).

**FIGURE 3:**
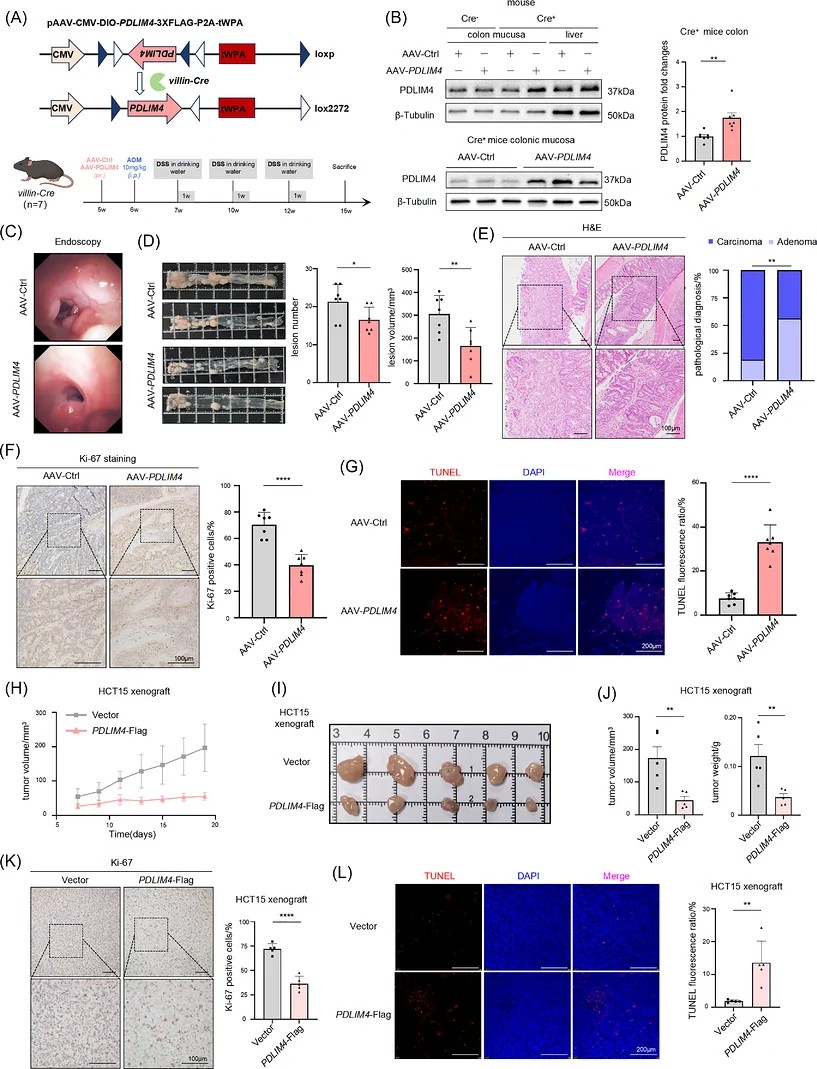
PDLIM4 overexpression suppresses colorectal tumour growth in vivo. (A) The schematic illustrates the generation of mice with intestinal mucosa‐specific PDLIM4‐overexpressing mice (AAV‐*PDLIM4*) and the subsequent AOM/DSS‐induced CRC model. (B) Western blot analysis detected the specific overexpression of PDLIM4 in colon tissue (*n* = 3 per group). (C) Representative images of colonoscopy at 15w. (D) Macroscopic views of the resected colons at sacrifice, accompanied by quantitative analyses of tumour number and volume in the AAV‐Ctrl and AAV‐*PDLIM4* cohorts (*n* = 7 mice per group). (E) Haematoxylin and eosin (H&E) staining and histopathological evaluation of the AOM/DSS‐induced colonic lesions (*n* = 7 for each group). Scale bars = 100 µm. (F and G) Representative images and corresponding quantitative analyses of Ki‐67 IHC staining (F) and TUNEL assay (G) in the colonic tumour tissues. Scale bars = 100 µm (left), 200 µm (right) (*n* = 7 for each group). (H) In vivo growth curve of subcutaneous xenografts established from control (Vector) and *PDLIM4*‐overexpressing (*PDLIM4*‐Flag) CRC cells (*n* = 5 mice per group). (I, J) Representative tumour images (I), tumour volumes and tumour weight (J) of *PDLIM4*‐overexpressing (*PDLIM4*‐Flag) and vector group. (K, L) Ki‐67 IHC staining (K) and TUNEL assay (L) of the xenograft tumour sections. Scale bars = 100 µm (Left), 200 µm (Right) (*n* = 5 for each group). Data are presented as mean ± SEM. ****p < .0001; ***p < .001; **p < .01; *p < .05; n.s. *p *> .05.

To further substantiate the anti‐tumour function of PDLIM4, we employed a human CRC cell xenograft model in nude mice. Western blot analysis confirmed markedly increased PDLIM4 expression in PDLIM4‐Flag xenograft tumours compared with vector controls (Figure S3D). In line with the AOM/DSS model findings, ectopic expression of PDLIM4 markedly suppressed tumour progression, as evidenced by significantly reduced tumour growth and lower terminal tumour weights compared with the control group (Figure 3H–J). Immunohistochemical analyses of the xenograft tissues further validated the anti‐proliferative (reduced Ki‐67) and pro‐apoptotic (increased TUNEL) effects of PDLIM4 in vivo (Figure 3K,L). Collectively, these findings from two in vivo models indicate that PDLIM4 restrains CRC progression by restricting tumour cell proliferation and facilitating apoptosis.

### 3.4 DNMT3A‐mediated promoter hypermethylation drives *PDLIM4* silencing in CRC

Building upon our observation of the inverse correlation between PDLIM4 expression and its promoter methylation status, we sought to delineate the underlying epigenetic regulatory mechanism. Pharmacological demethylation using 5‐Azacytidine (5‐AZA) increased *PDLIM4* mRNA expression in SW480, HCT116, Caco‐2, HT29, HCT15, and RKO cells, and increased PDLIM4 protein expression in HCT116, Caco‐2, HT29, HCT15, and RKO cells (Figure 4A–E; Figure S4A). Functionally, 5‐AZA treatment significantly inhibited cell proliferation in all six CRC cell lines (Figure S4C). These results further support the methylation‐dependent regulation of PDLIM4 expression in CRC cells.

**FIGURE 4:**
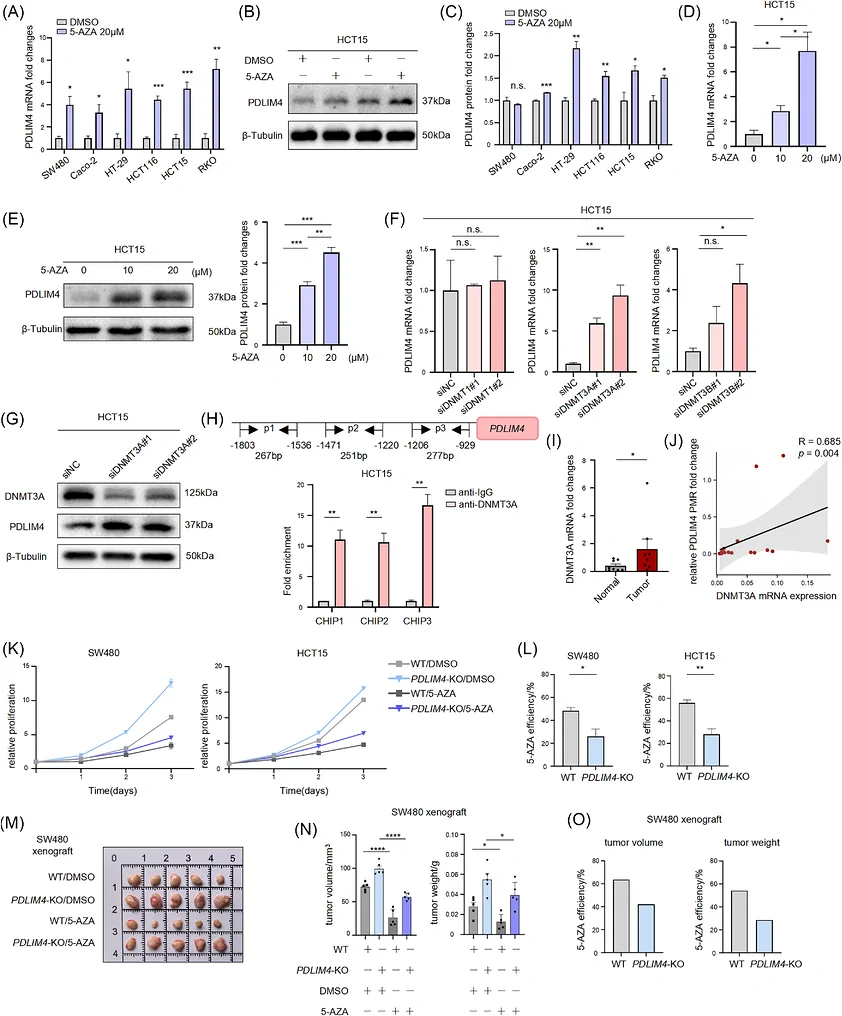
DNMT3A‐mediated promoter hypermethylation silences PDLIM4 and contributes to the anti‐tumour response to 5‐Azacytidine in CRC. (A) Quantitative RT‐PCR analyses demonstrating the restoration of PDLIM4 mRNA expression across a panel of CRC cell lines following pharmacological demethylation with 5‐Azacytidine (5‐AZA, 20 µM) (*n* = 3). (B) Representative image of western blot analyses showing the upregulation of PDLIM4 protein expression in HCT15 cells with 20 µM 5‐AZA. (C) Quantitative analyses of western blot images demonstrating the restoration of PDLIM4 protein expression across a panel of CRC cell lines following pharmacological demethylation with 20 µM 5‐AZA (*n* = 3). (D and E) Dose‐dependent re‐expression of PDLIM4 mRNA (D) and protein (E) in HCT15 cells subjected to increasing concentrations of 5‐AZA (*n* = 3). (F) Relative mRNA expression of PDLIM4 in HCT15 cells following the knockdown of DNMT1, DNMT3A, or DNMT3B (*n* = 3). (G) Western blot analysis confirming the specific upregulation of PDLIM4 protein upon DNMT3A depletion in HCT15 cells. (H) The endogenous interaction between DNMT3A and the *PDLIM4* promoter was evaluated by ChIP‒qPCR in HCT15 cells (*n* = 3). (I) Quantitative RT‐PCR analysis of DNMT3A mRNA expression in fresh CRC tissues (*n* = 8) and paired normal tissues (*n* = 8). (J) Scatter plots show the Spearman correlation between PDLIM4 methylation levels and DNMT3A mRNA expression in human colon tissue (*n* = 16). (K) CCK‐8 assays comparing the effects of 5‐AZA on cell viability in wild‐type (WT) and PDLIM4‐knockout (KO) SW480 and HCT15 cells (*n* = 3–4). (L) Efficiency of 5‐AZA in inhibiting proliferation in the indicated cell lines (*n* = 3). (M) Representative images of subcutaneous xenograft tumours established from wild‐type (WT) and PDLIM4‐knockout (*PDLIM4*‐KO) SW480 cells treated with 5‐AZA or DMSO (*n* = 5 mice per group). (N) Tumour volumes and tumour weight of subcutaneous xenografts in *PDLIM4*‐KO and WT groups (*n* = 5 mice per group). (O) Efficiency of 5‐AZA in inhibiting growth of subcutaneous xenografts established from WT and *PDLIM4*‐KO SW480 cells. Data are presented as mean ± SEM. ****p < .0001; ***p < .001; **p < .01; *p < .05; n.s. *p *> .05.

To identify the specific DNA methyltransferase (DNMT) orchestrating this epigenetic repression, we systematically silenced individual DNMT isoforms (Figure S4C). Knockdown of DNMT3A significantly restored PDLIM4 mRNA and protein expression, whereas depletion of DNMT1 or DNMT3B had little effect on PDLIM4 expression (Figure 4F,G). Furthermore, chromatin immunoprecipitation‐qPCR (ChIP‐qPCR) assays in HCT15 cells confirmed the direct endogenous recruitment and physical binding of DNMT3A to the *PDLIM4* promoter region (Figure 4H; Figure S4D). QPCR analysis of human colon biopsy specimens also revealed higher DNMT3A mRNA expression in CRC tissues than in adjacent normal tissues, together with a positive correlation between DNMT3A expression and PDLIM4 methylation levels (Figure 4I,J). Together, these data support DNMT3A as a major upstream epigenetic regulator of PDLIM4 silencing in CRC.

As DNA demethylating agents can reactivate epigenetically silenced tumour suppressor genes and produce anti‐tumour effects, we hypothesized that PDLIM4 contributes to the anti‐tumour effects of 5‐AZA. To test this, we subjected wild‐type and *PDLIM4*‐KO CRC cells to 5‐AZA treatment (Figure 4J). Strikingly, the genetic ablation of *PDLIM4* significantly blunted the growth‐inhibitory effects of 5‐AZA compared with the wild‐type controls (Figure 4K,L). To determine whether PDLIM4 also contributes to the anti‐tumour effect of 5‐AZA in vivo, we established subcutaneous xenografts in BALB/c nude mice using SW480 WT and *PDLIM4*‐KO cells. 5‐AZA treatment significantly reduced tumour volume and tumour weight in WT xenografts, whereas a weaker inhibitory effect was observed in PDLIM4‐KO tumours (Figure 4M,N). Consistently, the calculated inhibition efficiencies of 5‐AZA for both tumour volume and tumour weight were lower in PDLIM4‐KO xenografts than in WT xenografts (Figure 4O). These findings further support a contribution of PDLIM4 to the anti‐tumour activity of 5‐AZA in vivo.

Collectively, these findings support DNMT3A‐mediated promoter hypermethylation as an important mechanism of PDLIM4 silencing and indicate that PDLIM4 reactivation contributes to the anti‐tumour effects of 5‐AZA in CRC.

### 3.5 PDLIM4 exerts its tumour‐suppressive functions by physically interacting with and antagonizing BTF3

Since PDZ‐LIM family members classically serve as scaffolding proteins to orchestrate intracellular signal transduction, we hypothesized that PDLIM4 exerts its anti‐tumour effects via specific protein–protein interactions. To map its downstream interactome, co‐immunoprecipitation followed by mass spectrometry (Co‐IP/MS) was performed in RKO cells (Figure 5A). Based on fold‐change and score thresholds, several candidate interacting partners emerged, including PALLD, ENO2, ACTN1, BTF3, EIF1, and ACTN4. Given its well‐documented oncogenic roles in CRC and other malignancies, basic transcription factor 3 (BTF3) was prioritized for further investigation. Indeed, reciprocal Co‐IP and immunofluorescence (IF) co‐localization assays definitively confirmed a stable physical interaction between PDLIM4 and BTF3 in CRC cells (Figure 5B,C). To evaluate the clinical relevance of this interaction, we assessed BTF3 protein expression in clinical samples across the normal‐adenoma‐carcinoma sequence using IHC. In contrast to the progressive loss of PDLIM4, BTF3 expression increased from adenomas to carcinomas (Figure 5D). Subsequent correlation analysis confirmed a significant inverse relationship between PDLIM4 and BTF3 protein levels in clinical specimens (Figure 5E).

**FIGURE 5:**
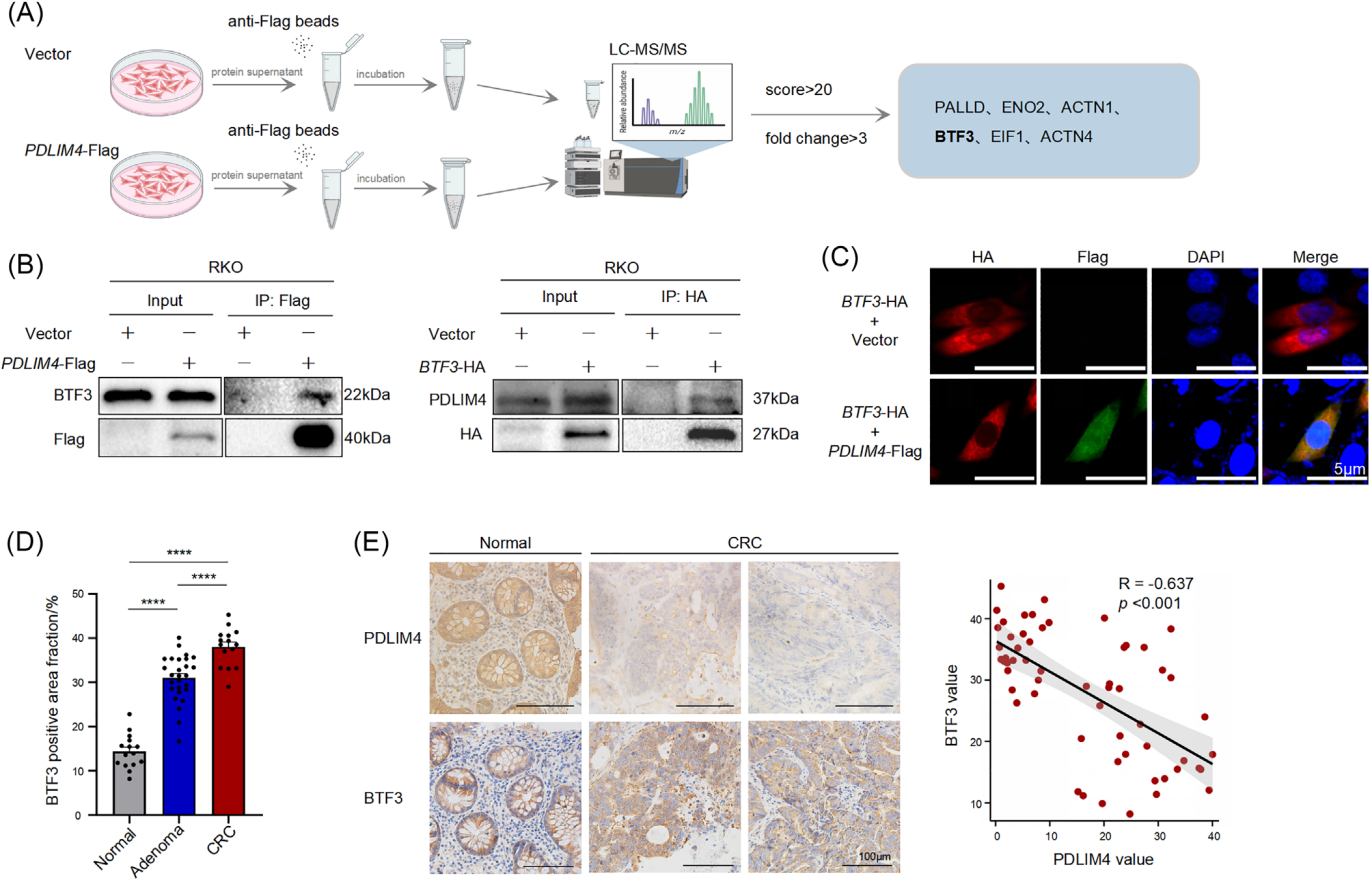
PDLIM4 interacts with BTF3 and shows an inverse expression pattern during CRC progression. (A) Schematic overview of the mass spectrometry (MS)‐based interactome screening strategy utilized to identify novel PDLIM4‐binding partners. (B, C) Co‐immunoprecipitation (Co‐IP) (B) and immunofluorescence (IF) analysis (C) confirming the interaction between PDLIM4 and BTF3. (D) IHC assessment of BTF3 protein levels across the normal (*n* = 15), adenoma (*n* = 29) and colorectal cancer (*n* = 15) tissues. (E) Representative IHC images of PDLIM4 and BTF3 in human colon tissues, with Spearman correlation analysis of their IHC expression levels (*n* = 59). Scale bars = 100 µm. Data are presented as mean ± SEM. ****p < .0001; ***p < .001; **p < .01; *p < .05; n.s. *p *> .05.

To determine whether BTF3 acts as the critical downstream effector mediating the malignant phenotypes induced by PDLIM4 loss, we performed genetic rescue experiments. We specifically depleted BTF3 using siRNA in both wild‐type (WT) and *PDLIM4*‐KO SW480 cells (Figure 6A). Consistent results from CCK‐8, EdU incorporation, and colony formation assays indicated that BTF3 knockdown attenuated the enhanced proliferative phenotype associated with PDLIM4 loss (Figure 6B–D). Furthermore, silencing BTF3 successfully reversed the apoptosis resistance (Figure 6E) and the enhanced sphere‐forming capacity (stemness) (Figure 6F) triggered by *PDLIM4* knockout. Collectively, these experiments support that PDLIM4 restrains CRC progression, at least in part, through inhibition of BTF3‐associated oncogenic signalling.

**FIGURE 6:**
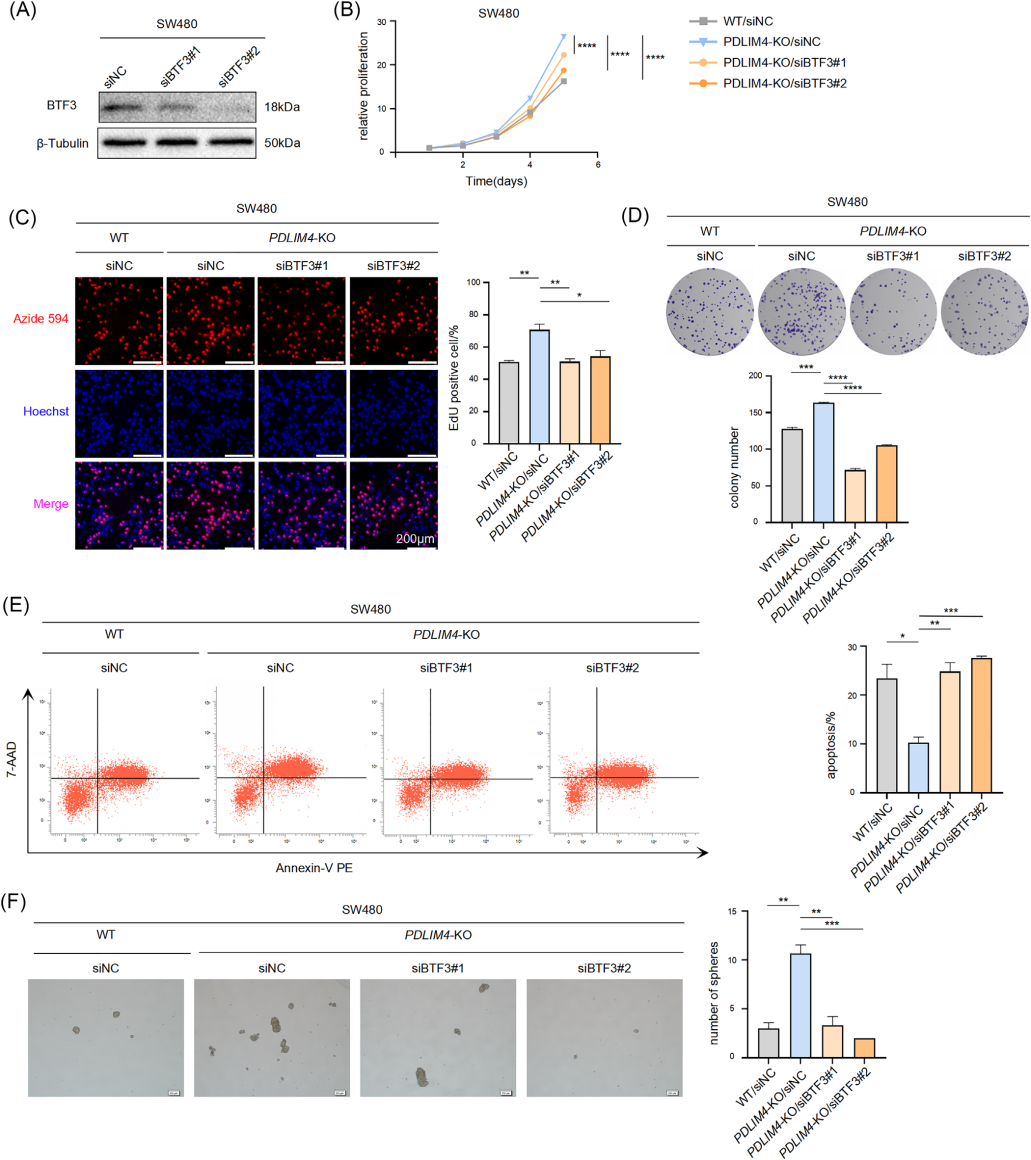
BTF3 depletion abrogates the oncogenic function conferred by PDLIM4 deficiency. (A) Western blot validation of BTF3 knockdown efficiency in SW480 cells. (B–D) Evaluation of cell proliferation via CCK‐8 assays (B), EdU staining (C) and colony formation assay (D) in *PDLIM4*‐KO SW480 and WT cells with or without knockdown of BTF3 (*n* = 3). Scale bars, 200 µm. (E) Flow cytometric analysis and quantification of H_2_O_2_‐induced apoptosis in wild‐type (WT) and PDLIM4‐knockout (*PDLIM4*‐KO) SW480 cells with or without BTF3 knockdown (*n* = 3). (F) Tumoursphere formation assay in wild‐type (WT) and PDLIM4‐knockout (*PDLIM4*‐KO) cells with or without BTF3 knockdown (*n* = 3). Scale bars = 200 µm. Data are presented as mean ± SEM. ****p < .0001; ***p < .001; **p < .01; *p < .05; n.s. *p *> .05.

### 3.6 PDLIM4 regulates BTF3 protein stability through autophagic‐lysosomal degradation

To elucidate the molecular mechanisms underlying the inverse relationship between PDLIM4 and BTF3, we first examined whether this regulation occurs at the transcriptional or post‐translational level. Quantitative RT‐PCR analysis revealed that neither the genetic ablation nor the overexpression of *PDLIM4* altered BTF3 mRNA levels (Figure 7A). In contrast, Western blot analysis demonstrated that *PDLIM4* depletion accumulated BTF3 protein, whereas *PDLIM4* overexpression diminished it (Figure 7B). This uncoupling of mRNA and protein dynamics suggested that PDLIM4 negatively regulates BTF3 via posttranslational protein stability. Indeed, classical cycloheximide (CHX) chase assays confirmed that *PDLIM4* knockout significantly prolonged the half‐life of BTF3 protein in SW480 cells, while *PDLIM4* overexpression accelerated its degradation rate in RKO cells (Figure 7C,D).

**FIGURE 7:**
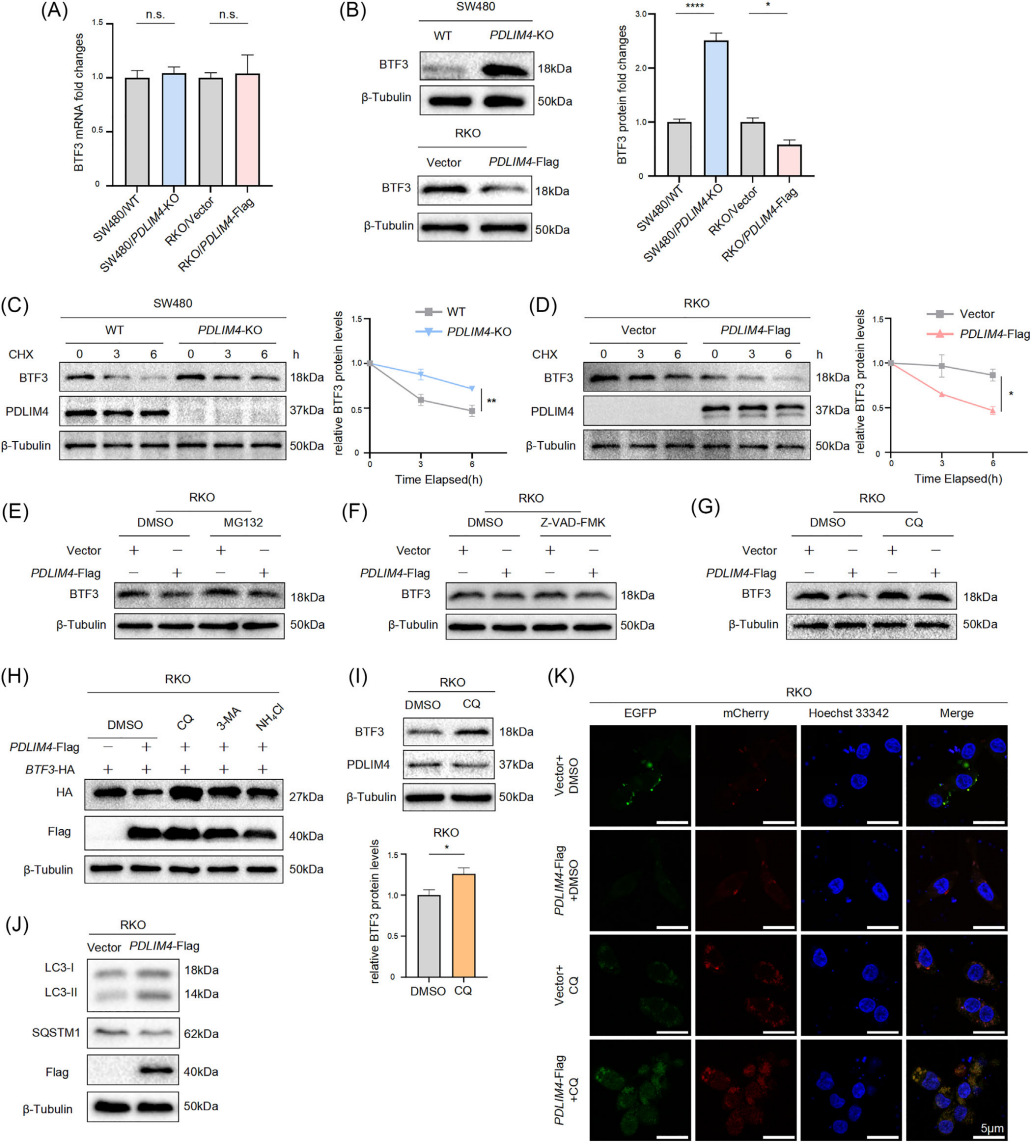
PDLIM4 promotes BTF3 degradation through the autophagy‐lysosome pathway. (A, B) mRNA and protein levels of BTF3 in *PDLIM4*‐KO SW480 and *PDLIM4*‐overexpressing RKO analyzed by qRT‐PCR (A) and WB (B) (*n* = 3). (C, D) Cycloheximide (CHX)‐chase assays evaluating the degradation of BTF3 protein in *PDLIM4*‐KO SW480 (C) and *PDLIM4*‐overexpressing RKO (*n* = 3) (D). (E–G) Western blot evaluation of BTF3 protein stability in *PDLIM4*‐overexpressing cells treated with the proteasome inhibitor MG132 (E), the pan‐caspase inhibitor Z‐VAD‐FMK (F), or the autophagy inhibitor chloroquine (CQ) (G). (H) Further validation of the BTF3 protein degradation route using autophagic‐lysosomal pathway inhibitors (CQ, 3‐Methyladenine [3‐MA] and NH_4_Cl) in *PDLIM4*‐overexpressing RKO (*n* = 3). (I) Western blot evaluation of BTF3 and PDLIM4 in RKO treated with CQ (20 µM, 48 h) (*n* = 3). (J) Western blot evaluation of autophagy marker LC3‐I/II and SQSTM1 in *PDLIM4*‐overexpressing RKO (*n* = 3). (K) Representative confocal fluorescence images tracking autophagic flux using the mCherry‐EGFP‐LC3 reporter system in *PDLIM4*‐overexpressing cells, treated with vehicle (DMSO) or CQ for 24 h (*n* = 3). Data are presented as mean ± SEM. ****p < .0001; ***p < .001; **p < .01; *p < .05; n.s. *p *> .05.

To determine the proteolytic pathway involved in PDLIM4‐mediated BTF3 degradation, PDLIM4‐overexpressing cells were treated with MG132, Z‐VAD‐FMK, or chloroquine (CQ) to inhibit proteasomal, caspase‐dependent, or autophagic‐lysosomal degradation, respectively. PDLIM4‐induced BTF3 degradation was reversed by CQ treatment, whereas MG132 and Z‐VAD‐FMK had little effect (Figure 7E–G). To validate this autophagic‐lysosomal dependency, we co‐expressed PDLIM4 and BTF3 in RKO cells and treated them with two additional well‐characterized lysosomal inhibitors, 3‐methyladenine (3‐MA) and ammonium chloride (NH_4_Cl). Consistent with the CQ results, both 3‐MA and NH_4_Cl effectively restored BTF3 protein levels (Figure 7H). Having established that PDLIM4 promotes BTF3 degradation via the autophagy‐lysosome pathway, we next examined whether PDLIM4 itself is subject to autophagic regulation. CQ treatment resulted in accumulation of BTF3, whereas PDLIM4 protein levels remained unchanged (Figure 7I), suggesting that PDLIM4 itself is not substantially degraded through the autophagy‐lysosome pathway under these conditions. We further examined whether PDLIM4 affects autophagy‐related activity. PDLIM4 overexpression increased the LC3‐II/LC3‐I ratio and the number of mCherry‐EGFP‐LC3 fluorescent puncta in CRC cells, supporting enhanced autophagic flux (Figure 7J,K). Collectively, these findings suggest that PDLIM4 limits BTF3 accumulation by promoting its degradation through the autophagy‐lysosome pathway.

### 3.7 PDLIM4 restrains CRC progression by abrogating the BTF3‐driven activation of the PI3K/Akt signalling pathway

To unearth the downstream oncogenic signalling networks hyperactivated by *PDLIM4* depletion, we performed transcriptome sequencing (RNA‐seq) on *PDLIM4*‐KO and WT SW480 cells. Kyoto Encyclopedia of Genes and Genomes (KEGG) enrichment analysis strikingly revealed that the PI3K/Akt signalling cascade was the most prominently enriched pathway in *PDLIM4*‐deficient cells (Figure 8A). Corroborating our in vitro profiling, pathway enrichment analysis of the top 200 genes closely correlated with *PDLIM4* in the TCGA‐CRC clinical dataset independently converged on the PI3K/Akt signalling pathway (Figure 8B), providing clinical relevance to our genomic findings. Consistent with the transcriptome data, Western blot analysis validated that the levels of active, phosphorylated AKT (p‐AKT) were elevated following *PDLIM4* knockout, but diminished upon ectopic *PDLIM4* overexpression (Figure 8C,D). To examine the role of BTF3 in PI3K/Akt signalling, we overexpressed *BTF3* in CRC cells and observed increased AKT phosphorylation (Figure 8E). More importantly, genetically silencing *BTF3* distinctly abolished the aberrant PI3K/Akt hyperactivation induced by *PDLIM4* deletion (Figure 8F). Analysis of tumour tissues from both AAV‐PDLIM4 mice and subcutaneous xenografts further showed reduced BTF3 expression and p‐AKT levels following PDLIM4 overexpression, consistent with the in vitro findings (Figure S5A,B). To further investigate how BTF3 regulates PI3K/Akt signalling, we re‐analyzed the RNA‐seq data from *PDLIM4‐*KO SW480 and screened differentially expressed genes involved upstream of the PI3K/Akt pathway (Figure S5C). Candidate genes were then examined by qRT‐PCR in *BTF3*‐overexpressing SW480 cells. Among these candidates, Neurotrophic receptor tyrosine kinase 2 (NTRK2) was significantly upregulated following *BTF3* overexpression (Figure S5D). ChIP‐qPCR further demonstrated direct binding of BTF3 to the NTRK2 promoter (Figure S5E). These findings suggest that the PDLIM4/BTF3 axis may promote PI3K/Akt activation through transcriptional upregulation of NTRK2.

**FIGURE 8:**
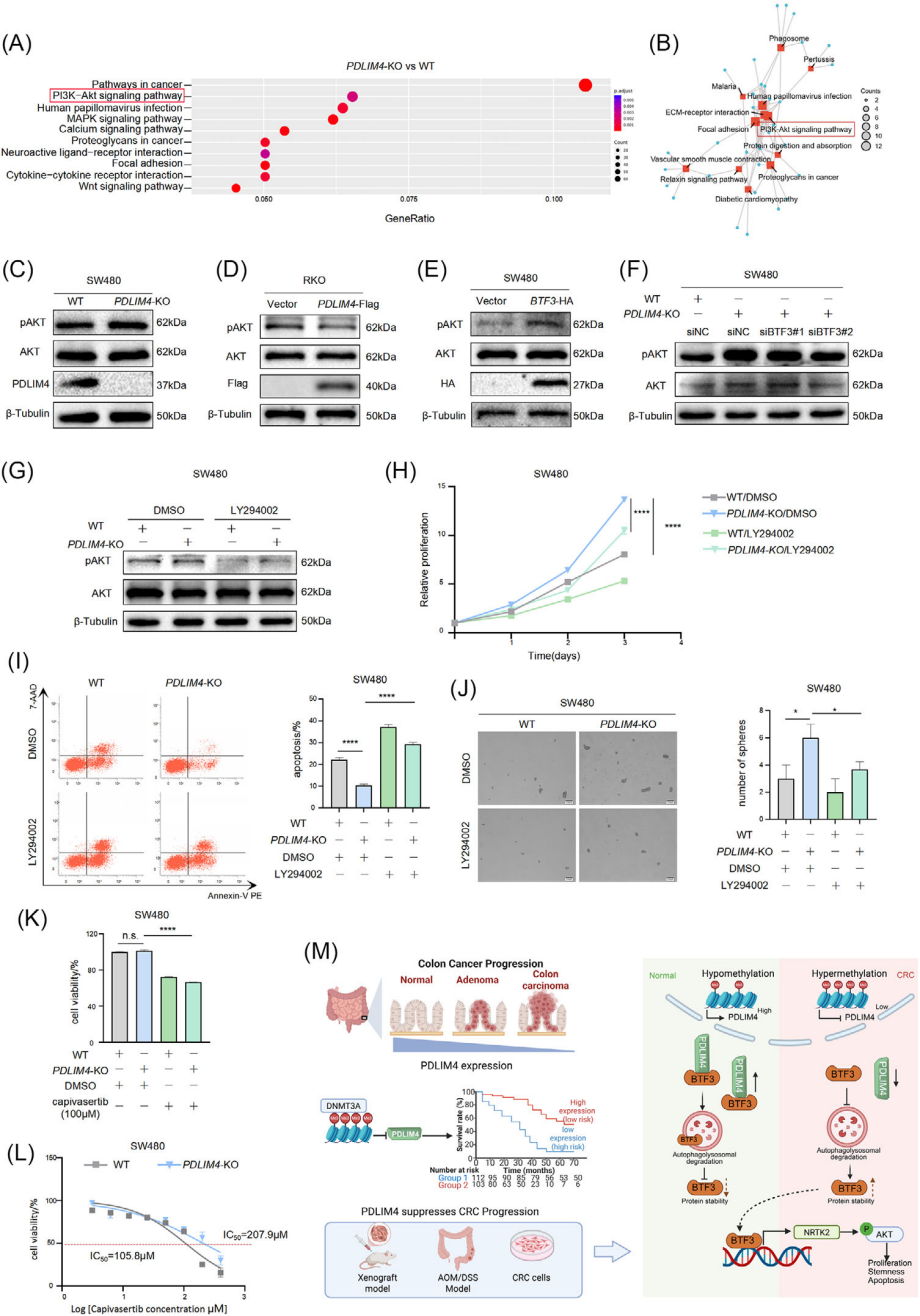
PDLIM4 suppresses CRC progression by restraining BTF3‐mediated PI3K/Akt signalling. (A and B) KEGG pathway enrichment analysis of differentially expressed genes between PDLIM4‐knockout (*PDLIM4*‐KO) and wild‐type (WT) SW480 cells (A), and the top 200 genes most correlated with *PDLIM4* in the TCGA‐CRC cohort (B). (C–E) Western blot analysis of phosphorylated AKT (pAKT) and total AKT in *PDLIM4*‐KO SW480 cells (C), *PDLIM4*‐overexpressing RKO cells (D), *BTF3*‐overexpression SW480 cells (E) and respective controls (*n* = 3). (F) Western blot analysis of pAKT and AKT in *PDLIM4*‐KO and WT SW480 cells with or without knockdown of BTF3 (*n* = 3). (G) Western blot confirming the pharmacological blockade of PI3K/Akt signalling in *PDLIM4*‐KO and WT cells using the specific PI3K inhibitor LY294002. (H–J) CCK‐8 assays (H), flow cytometric analysis of H_2_O_2_‐induced apoptosis (I), and tumoursphere formation assays (J) in wild‐type (WT) and PDLIM4‐knockout (PDLIM4‐KO) SW480 cells treated with DMSO or LY294002 (*n* = 4). (K) CCK8 assay of survival rate in *PDLIM4*‐KO and WT SW480 cells treated with 100 µM capivasertib for 48 h (*n* = 4). (L) Detection of the half‐maximal inhibitory concentration (IC50) of capivasertib on *PDLIM4*‐KO and WT SW480 cells at 48 h using the CCK8 assay (*n* = 4). (M) Graphical abstract illustrating the DNMT3A–PDLIM4–BTF3–PI3K/Akt axis in colorectal cancer. DNMT3A‐mediated promoter hypermethylation silences PDLIM4, reduces BTF3 autophagic‐lysosomal degradation, and enhances PI3K/Akt signalling, thereby promoting colorectal tumorigenesis. (Created with BioRender.com). Data are presented as mean ± SEM. ****p < .0001; ***p < .001; **p < .01; *p < .05; n.s. *p *> .05.

To functionally verify whether the malignant phenotypes driven by *PDLIM4* loss are dependent on PI3K/Akt hyperactivation, we treated *PDLIM4*‐KO SW480 cells with LY294002, a specific PI3K/Akt inhibitor (Figure 8G). Pharmacological blockade of this pathway successfully neutralized the hyper‐proliferative advantage and enhanced stemness conferred by *PDLIM4* loss, while restoring their susceptibility to apoptosis (Figure 8H–J). To further validate the involvement of AKT signalling, we treated WT and *PDLIM4*‐KO cells with capivasertib (a structurally distinct AKT inhibitor). Like LY294002, capivasertib attenuated the increased proliferation associated with PDLIM4 loss (Figure 8K). However, dose‐response analysis showed that *PDLIM4*‐KO cells had a higher IC50 for capivasertib than WT cells (207.9 µM vs. 105.8 µM), indicating reduced rather than increased sensitivity to AKT inhibition (Figure 8L).

Together, these findings support a molecular pathway in which epigenetic silencing of PDLIM4 reduces BTF3 autophagic degradation, leading to increased BTF3 accumulation and enhanced NTRK2/PI3K/Akt signalling during CRC progression (Figure 8M).

## 4 DISCUSSION

The transition from benign adenoma to malignant carcinoma represents a critical window for early intervention in colorectal cancer (CRC). However, the specific epigenetic alterations underlying this early transformation remain incompletely understood.^25^ In the present study, we demonstrate that the DNA hypermethylation‐driven silencing of *PDLIM4* is a crucial early‐stage epigenetic event in CRC progression. By integrating methylation and transcriptome datasets with validation in both patient samples and mouse models, we show that the progressive loss of PDLIM4 correlates with the adenoma‐to‐carcinoma sequence and predicts adverse patient outcomes. Mechanistically, our study identifies a regulatory axis in which PDLIM4 interacts with BTF3 and promotes its autophagic‐lysosomal degradation, thereby restraining PI3K/Akt signalling. Collectively, these findings identify *PDLIM4* as a key epigenetic regulator in the intestinal epithelium, providing mechanistic insights into early tumorigenesis and highlighting a potential target for diagnostic and therapeutic strategies.

Previous studies indicated that PDLIM4 exhibits context‐dependent roles in human malignancies. In gastric cancer, PDLIM4 has been reported to promote malignant progression and cisplatin resistance through competitive interaction with STUB1, thereby reducing HSP70 degradation and activating MAPK signalling.^26^ Conversely, PDLIM4 exhibits tumour‐suppressive effects in ovarian cancer by inactivating STAT3 signalling.^27^ In our present study, PDLIM4 acts as a tumour suppressor in CRC through regulation of the BTF3–PI3K/Akt axis. These distinct effects suggest that the function of PDLIM4 may be determined by tissue‐specific interacting partners, downstream signalling networks, and cellular context.

Moreover, we further identified promoter hypermethylation as an important mechanism underlying PDLIM4 silencing and showed that DNMT3A contributes to this epigenetic regulation. Although our data demonstrate DNMT3A binding to the PDLIM4 promoter and its role in PDLIM4 silencing, it remains to be determined whether DNMT3A directly catalyzes CpG methylation at this locus through its intrinsic methyltransferase activity, or whether additional cofactors, including DNMT3L, transcription factors, or other chromatin‐associated proteins, are required for its recruitment or catalytic activity.^19^, ^28^ Moreover, individual depletion of DNMT1 or DNMT3B did not appreciably restore PDLIM4 expression; however, potential functional compensation among DNMT isoforms following DNMT3A depletion cannot be excluded.

Consistent with the functional importance of this epigenetic regulation, restoration of PDLIM4 expression–either through genetic manipulation or pharmacological demethylation with 5‐AZA—effectively suppressed CRC progression by inhibiting proliferation and stemness while enhancing apoptosis. DNA methyltransferase inhibitors, such as decitabine, are currently used primarily in haematological malignancies, whereas their application in solid tumours remains under active investigation.^29^ Our findings therefore raise the possibility that PDLIM4 methylation may have value not only as a biomarker for early CRC detection but also as a marker associated with response to demethylating therapy. However, its predictive value will require further validation in preclinical models and clinical cohorts.

Beyond elucidating its epigenetic upstream regulation, our study delineates a distinct downstream mechanism for PDLIM4. Specifically, we show that epigenetic loss of PDLIM4 in CRC impairs the autophagic‐lysosomal degradation of BTF3. BTF3 was initially identified as a transcription factor that forms a complex with RNA polymerase II. Elevated expression has been reported across several cancer types, such as liver, colorectal, and gastric cancers.^30^, ^31^, ^32^ In addition to regulating the transcription of downstream genes, BTF3 has been reported to participate in tumour progression through protein interactions. For instance, BTF3 stabilizes BMI1 to maintain cancer stemness in colorectal cancer and prostate cancer,^23^, ^24^ and interacts with FOXM1 to enhance cell proliferation and glycolysis in hepatocellular carcinoma cells.^33^ Given its critical role in tumour progression, the precise regulation of BTF3 protein stability is essential for cellular homeostasis. Previous mechanistic studies governing BTF3 stability have primarily focused on the ubiquitin‐proteasome system; for instance, the deubiquitinase USP7 prevents BTF3 ubiquitination to maintain the malignant phenotype in hepatocellular carcinoma.^34^ Herein, we identify an alternative, previously unrecognized proteolytic route for BTF3 clearance. Our data show that PDLIM4 interacts with BTF3 and promotes its degradation through the autophagy‐lysosome pathway, representing a distinct mechanism from the ubiquitin‐proteasome‐mediated regulation of BTF3 reported in other cancer models. PDZ‐LIM family proteins are generally known for their roles as structural and signalling scaffolds. Our findings suggest that PDLIM4 may also participate in the regulation of protein turnover.

PI3K/Akt signalling is commonly dysregulated in cancer and contributes to malignant progression and resistance to anti‐cancer therapy. In CRC, this pathway has also been implicated in multiple aspects of tumour biology, including regulation of the tumour microenvironment.^35^ Epigenetic alterations can further modulate this pathway; for example, silencing of the tumour suppressor PTEN has been associated with enhanced PI3K/Akt activation in gastric and colorectal cancers.^36^, ^37^ Our findings suggest that PDLIM4 loss stabilizes BTF3, which in turn upregulates NTRK2, encoding the TrkB receptor, thereby providing a potential mechanism for activation of the PI3K/Akt pathway.^38^ Functionally, both LY294002 and capivasertib attenuated the proliferative phenotype associated with PDLIM4 loss, while LY294002 also reduced the enhanced stemness. However, dose‐response analysis showed that *PDLIM4*‐KO cells had a higher IC50 for capivasertib than WT cells, indicating that PDLIM4 loss did not confer increased sensitivity to AKT inhibition. These findings support a functional role of PI3K/Akt signalling downstream of the PDLIM4–BTF3 axis but also indicate that enhanced pathway activation does not necessarily predict greater pharmacological sensitivity to AKT inhibitors. Whether PDLIM4 or BTF3 status can predict response to PI3K/Akt‐targeted therapy remains uncertain and requires further investigation.

Several limitations of this study should be acknowledged. First, although our preliminary stool DNA analysis supports the detectability of PDLIM4 hypermethylation in a non‐invasive sample, the clinical validation remains limited by the relatively small sample size and the lack of an independent external validation cohort. Its diagnostic performance for adenoma and early CRC also remains to be established. Further validation in larger, independent prospective cohorts using faecal DNA or circulating tumour DNA (ctDNA) will be required.^39^, ^40^ Second, although our data demonstrate that PDLIM4 interacts with BTF3 and promotes its autophagic‐lysosomal degradation, the molecular mechanism linking this complex to the autophagy machinery remains unclear. It remains to be determined whether PDLIM4 directly interacts with LC3/GABARAP family proteins, which mediate cargo recruitment to autophagosomal membranes,^41^ or instead acts through selective autophagy receptors such as p62/SQSTM1 or NBR1.^42^ In addition, the structural domains responsible for the PDLIM4–BTF3 interaction have not yet been defined. Further interaction and domain‐mapping studies will be required to clarify how PDLIM4 facilitates the autophagic degradation of BTF3. Third, although BTF3 overexpression increased NTRK2 expression and ChIP‐qPCR demonstrated enrichment of BTF3 at the NTRK2 promoter, direct transcriptional activation of NTRK2 by BTF3 has not yet been confirmed by promoter‐reporter assays. Further luciferase reporters and loss‐of‐function studies will be required to determine whether NTRK2 is necessary for BTF3‐mediated activation of the PI3K/Akt pathway. Fourth, our in vivo studies focused primarily on tumour burden and tumour cell‐intrinsic phenotypes. Long‐term survival and tumour‐associated inflammatory changes were not assessed and warrant further investigation.

## 5 CONCLUSION

In this study, we identified DNA hypermethylation‐mediated silencing of PDLIM4 as an early epigenetic event in colorectal tumorigenesis. Reduced PDLIM4 expression promotes proliferation and stemness while suppressing apoptosis. Mechanistically, PDLIM4 promotes BTF3 autophagic‐lysosomal degradation and restrains PI3K/Akt signalling. These findings highlight the PDLIM4–BTF3–PI3K/Akt axis as an important regulatory pathway in CRC.

## AUTHOR CONTRIBUTIONS

Jiaxing Wang, Nan Zhang, and Si Liu designed the experiments. Jiaxing Wang, Boshen Lin, and Tong Ye performed the in vitro experiments. Jiaxing Wang, Songyuan Hou and Si Liu conducted the in vivo experiments. Jiaxing Wang, Chuan‐Guo Guo, Lingyu Qi and Ye Zong helped with the data analysis. Shutian Zhang, Peng Li and Si Liu provided funding. Jiaxing Wang, Nan Zhang and Si Liu wrote the manuscript. Jiaxing Wang, Nan Zhang, Peng Li and Si Liu revised and approved the manuscript. All authors have read and approved the final manuscript.

## CONFLICT OF INTEREST STATEMENT

The authors declare no conflicts of interest.

## CONSENT FOR PUBLICATION

All the authors are aware of and agree to the content of the paper and their being listed as co‐authors of the paper.

## ETHICS STATEMENT AND CONSENT TO PARTICIPATE

This study was approved by the Institutional Ethics Committee of Beijing Friendship Hospital (approval number 2018‐P2‐058‐05). All animal experiments were conducted in accordance with institutional guidelines and were approved by the Ethics Committee of Capital Medical University (approval number AEEI‐2025‐1130).

## Supporting information



Supporting Information

Supporting Information

## Data Availability

The RNA sequencing data generated in this study have been uploaded to the database (https://www.ncbi.nlm.nih.gov/sra/) under the accession number RJNA1356465. All other data supporting the findings are available from the corresponding author upon reasonable request.
