# Supplementary material for: Epigenetic silencing of PDLIM4 promotes colorectal cancer by impairing BTF3 autophagic degradation and activating PI3K/Akt signalling
Source: Clin Transl Med. 2026 Oct 30;16(11):e70882. doi: 10.1002/ctm2.70882 (PMC13720898; doi:10.1002/ctm2.70882)
Supplement: Supplementary file 1 [file CTM2-16-e70882-s001.docx]

**Figure S1. *PDLIM4* is a key gene in the development of CRC.** (A) Spearman correlation analysis between promoter methylation and gene expression (FPKM) of candidate genes using the DNA Methylation Interactive Visualization Database (DNMIVD). (B) Overall survival was stratified by high and low expression levels of ZNF415 in colorectal cancer (CRC). (C) Comparison of *PDLIM4* promoter methylation levels between primary CRC tumors and adjacent normal tissues, utilizing the UALCAN (The University of Alabama at Birmingham Cancer data analysis Portal) database. (D) Schematic diagram of AOM/DSS induced adenoma and CRC model in C57BL/6J mice (n=6 for each group). (E) Representative H&E staining illustrating the sequential histopathological changes in the murine colon throughout the AOM/DSS induction course. Scale bars, 100μm. (F and G) Quantitative RT-PCR (F) and Western blot (G) analyses of PDLIM4 respectively in human colorectal normal epithelial cells (NCM460) and six CRC cell lines (n =3). (H) Q-MSP analysis of *PDLIM4* methylation status across the NCM460 and CRC cell lines (n =3). COAD, colon adenocarcinoma; READ, rectum adenocarcinoma; AOM, azoxymethane; DSS, dextran sulfate sodium salt. Data are presented as mean ± SEM. **** *p*<0.0001; *** *p*<0.001; ** *p*<0.01; * *p*<0.05; n.s. *p*>0.05.

**Figure S2. PDLIM4 suppresses proliferation of HCT15 colorectal cancer cells in vitro.** (A) Western blot analysis confirmed *PDLIM4* knockout (*PDLIM4*-KO) and overexpression (*PDLIM4*-Flag) in HCT15 cells. (B-D) Evaluation of cell proliferation utilizing CCK-8 assays(B), EdU staining (C) and colony formation assay (D) in HCT15 following *PDLIM4* knockout or overexpression (n =3). Scale bars, 200μm. Data are presented as mean ± SEM. **** *p*<0.0001; *** *p*<0.001; ** *p*<0.01; * *p*<0.05; n.s. *p*>0.05.

**Figure S3. PDLIM4 overexpression reduces proliferation and promotes apoptosis in colorectal tumors.** (A) Representative images of PDLIM4 IHC staining in the colonic tumor tissues of mice in the AAV-Ctrl and AAV-*PDLIM4* cohorts. The colon of mice was photographed in three segments from ileocecus to anus, and the expression of PDLIM4 protein was detected by immunohistochemistry (n = 7 per group). Scale bars, 200μm. (B-C) Scatter plots show the correlation between PDLIM4 expression with Ki-67 proliferative index (B) and TUNEL assay (C). (D) Western blot analysis confirming PDLIM4 overexpression in tumor xenografts derived from control (Vector) and PDLIM4-overexpressing (PDLIM4-Flag) HCT15 cells (n = 5 per group). Data are presented as mean ± SEM. **** *p*<0.0001; *** *p*<0.001; ** *p*<0.01; * *p*<0.05; n.s. *p*>0.05.

**Figure S4. Validation of the epigenetic regulation of *PDLIM4* and the anti-proliferative efficacy of 5-Azacytidine in CRC cells.** (A) Western blot analysis of PDLIM4 protein levels across multiple CRC cell lines following pharmacological demethylation with 5-Azacytidine (5-AZA, 20 μM) relative to the vehicle (DMSO) contro. (B) CCK-8 cell viability assays evaluating the anti-proliferative impact of 5-AZA (20 μM on the corresponding CRC cell lines (n = 4). (C) Quantitative RT-PCR analysis showed DNMT1, DNMT3A and DNMT3B knockdown efficiency in HCT15 cells (n = 3). (D) Nucleic acid gel electrophoresis revealed the endogenous interaction between DNMT3A and the promoter of PDLIM4 in HCT15 cells. Data are presented as mean ± SEM. **** *p*<0.0001; *** *p*<0.001; ** *p*<0.01; * *p*<0.05; n.s. *p*>0.05.

**Figure S5. BTF3 activates the PI3K-AKT pathway by promoting NTRK2 transcription.** (A and B) Western blot analysis of the PDLIM4–BTF3–PI3K/Akt axis in AOM/DSS-induced tumors with intestinal epithelial-specific PDLIM4 overexpression (A) and PDLIM4-overexpressing xenograft tumors (B). (C) KEGG pathway map of PI3K/Akt signaling showing differentially expressed genes in PDLIM4-knockout SW480 cells compared with wild-type cells. Upregulated genes are shown in red and downregulated genes in green. (D) Quantitative RT-PCR analysis of candidate gene expression following BTF3 overexpression in SW480 cells (n = 3). (E) ChIP-qPCR was performed to assess BTF3 enrichment at the NTRK2 promoter in SW480 cells (n = 3). Data are presented as mean ± SEM. **** *p*<0.0001; *** *p*<0.001; ** *p*<0.01; * *p*<0.05; n.s. *p*>0.05.
