# Supplementary material for: Epigenetic silencing of PDLIM4 promotes colorectal cancer by impairing BTF3 autophagic degradation and activating PI3K/Akt signalling
Source: Clin Transl Med. 2026 Oct 30;16(11):e70882. doi: 10.1002/ctm2.70882 (PMC13720898; doi:10.1002/ctm2.70882)
Supplement: Supplementary file 2 [file CTM2-16-e70882-s002.docx]

Supplementary Table 1. Primer sequences for MSP and Q-MSP.

| Species | Targets | Primer sequences |
| --- | --- | --- |
| Human | PDLIM4-M-Forward | GTGTCGGGTTGTCGGTAGTC |
|  | PDLIM4-M-Reverse | CGCTCCCTATACGCCTAAATTAAC |
|  | input control-Forward | TGTGATGGAGGAGGTTTAGTAAGT |
|  | input control-Reverse | AACCAATAAAAACCTACTCCTCCCTTAA |
| Mouse | PDLIM4-M-Forward | GGTTAGAGTATCGGTTCGTAAAAGC |
|  | PDLIM4-M-Reverse | TTTCCCAAACTAAACCCCGCA |
|  | PDLIM4-U-Forward | GGGTTAGAGTATTGGTTTGTAAAAGT |
|  | PDLIM4-U-Reverse | TTTCCCAAACTAAACCCCACA |

Supplementary Table 2. Antibodies list.

| Target | Application | Brand | Catalog number |
| --- | --- | --- | --- |
| PDLIM4 | WB | Proteintech | 18543-1-AP |
| PDLIM4 | IHC | Abcam | ab251701 |
| Ki-67 | IHC | Proteintech | 28074-1-AP |
| DNMT1 | WB | Proteintech | 24206-1-AP |
| DNMT3A | WB, CHIP | Proteintech | 20954-1-AP |
| DNMT3B | WB | Proteintech | 26971-1-AP |
| Bax | WB | Proteintech | 50599-2-Ig |
| Bcl-2 | WB | Proteintech | 68103-1-Ig |
| Cleaved caspase 3 | WB | Proteintech | 68773-1-Ig |
| Caspase 3 | WB | Proteintech | 19677-1-AP |
| CD133 | WB | Proteintech | 18470-1-AP |
| OCT4 | WB | Proteintech | 60242-1-Ig |
| Nanog | WB | Proteintech | 14295-1-AP |
| PALLD | WB,IP | Proteintech | 10853-1-AP |
| ENO2 | WB | Proteintech | 10149-1-AP |
| BTF3 | WB | Abcam | ab203517 |
| BTF3 | IHC, CHIP | Abmart | TN23659 |
| LC3 | WB | Proteintech | 14600-1-AP |
| SQSTM1/p62 | WB | Proteintech | 66184-1-Ig |
| Phospho-AKT (Ser473) | WB | Proteintech | 66444-1-Ig |
| AKT | WB | Proteintech | 10176-2-AP |
| Flag | WB | Abmart | M20026 |
| Flag | IF | Proteintech | 66008-4-Ig |
| HA | WB | Abmart | M20021 |
| HA | IF | Proteintech | 81290-1-RR |
| β-Tubulin | WB | Proteintech | 66240-1-lg |
| Mouse IgG | CHIP | Proteintech | B900620 |
| Rabbit IgG Isotype Control | CHIP | Proteintech | 98136-1-RR |

Supplementary Table 3. Small interfeing RNA (siRNA) used in this study.

| Target | sequence |  |
| --- | --- | --- |
| DNMT1 | siRNA-1 sense | CAAUGAGACUGACAUCAAAdTdT |
|  | siRNA-1 antisense | UUUGAUGUCAGUCUCAUUGdTdT |
|  | siRNA-2 sense | GGAAGUGAAUGGACGUCUAdTdT |
|  | siRNA-2 antisense | UAGACGUCCAUUCACUUCCdTdT |
| DNMT3A | siRNA-1 sense | GGGUUGGACAUCAUCUCCUGdTdT |
|  | siRNA-1 antisense | CAGGAGAUGAUGUCCAACCCdTdT |
|  | siRNA-2 sense | CCAGAUGUUCUUCGCUAAUdTdT |
|  | siRNA-2 antisense | AUUAGCGAAGAACAUCUGGdTdT |
| DNMT3B | siRNA-1 sense | AGAUGACGGAUGCCUAGAGdTdT |
|  | siRNA-1 antisense | CUCUAGGCAUCCGUCAUCUdTdT |
|  | siRNA-2 sense | GCUCUUACCUUACCAUCGAdTdT |
|  | siRNA-2 antisense | UCGAUGGUAAGGUAAGAGCdTdT |
| BTF3 | siRNA-1 sense | GCCGAAGAAGCCUGGGAAUCAdTdT |
|  | siRNA-1 antisense | UGAUUCCCAGGCUUCUUCGGCdTdT |
|  | siRNA-2 sense | GCAGGCACAAGUGCGCAUUdTdT |
|  | siRNA-2 antisense | AAUGCGCACUUGUGCCUGCdTdT |

Supplementary Table 4. Primer sequences for qRT-PCR.

| Gene | Forward primer | Reverse primer |
| --- | --- | --- |
| PDLIM4 | TGCCACGATCACCTCACACTG | GCCGCCTGCTGGTTGTTG |
| DNMT1 | AGAACGGTGCTCATGCTTACA | CTCTACGGGCTTCACTTCTTG |
| DNMT3A | CCGATGCTGGGGACAAGAAT | CCCGTCATCCACCAAGACAC |
| DNMT3B | GGCAAGTTCTCCGAGGTCTCTG | TGGTACATGGCTTTTCGATAGGA |
| CD133 | GGTGCTCTTCCAGGAACCTC | GGAAACTCTCTCGGTCCAGC |
| OCT4 | CTTGCTGCAGAAGTGGGTGGAGGAA | CTGCAGTGTGGGTTTCGGGCA |
| Nanog | AATACCTCAGCCTCCAGCAGATG | TGCGTCACACCATTGCTATTCTTC |
| BTF3 | GCCAGTCTCCTTAAACTAGTCAG | TTTCACCATTACAGGCCATGCT |
| CDC37 | AACACAAGACCTTCGTGGAAAA | TAATTGGCTGTCTCCTCGCAC |
| COL6A | ACAGTGACGAGGTGGAGATCA | GATAGCGCAGTCGGTGTAGG |
| COL9A | TCCCTGGTGAGATTGGAATCC | TTGGTTGGACACAGGAAATCC |
| ERBB3 | GGTGATGGGGAACCTTGAGAT | CTGTCACTTCTCGAATCCACTG |
| LPAR1 | GCTGCCATCTCTACTTCCATC | AAGCGGCGGTTGACATAGATT |
| NTRK1 | GTCAGCCACGGTGATGAAATC | CAGCACGTCACGTTCTTCCT |
| NTRK2 | ACCCGAAACAAACTGACGAGT | AGCATGTAAATGGATTGCCCA |
| PGF | CATGTTCAGCCCATCCTGTG | ACCTTTCCGGCTTCATCTTC |
| GAPDH | GGAGCGAGATCCCTCCAAAAT | GGCTGTTGTCATACTTCTCATGG |

Supplementary Table 5. Primer sequences for CHIP.

| Gene | Primer sequences |
| --- | --- |
| PDLIM4-P1 | F-CAGGATGGAGGCAAGGAGTG |
|  | R-GTAAGGGGCAGCCATCACTT |
| PDLIM4-P2 | F-AATGCAGTGACCAGCTCTCC |
|  | R-TGTAGGAGGCAAGATGCTGC |
| PDLIM4-P3 | F-TCTACTCTGAAGGGCCCCTC |
|  | R-CACCAACCTCCCTCTGGTTC |
| NTRK2-P1 | ATTCCCGCGGTTACACTCAG |
|  | GCAGAGTTTGGGGATGGGAA |
| NTRK2-P2 | CTGACAAGACAGGCGGAGTT |
|  | AACGGCCAGGCTGAAGTTTA |
| NTRK2-P3 | CTCTGGCCCAGTACTGAAGC |
|  | TTCCTTGCGTTCTGAGTGCT |
